# Bioassays in Allergy: Technical Characteristics, Limitations, and Pathways to Clinical Implementation—An EAACI Position Paper

**DOI:** 10.1002/clt2.70210

**Published:** 2026-09-30

**Authors:** Cagatay Karaaslan, Oscar Palomares, Busra Kilic, Cristobalina Mayorga, Hans Jürgen Hoffmann, Bernadette Eberlein, Edward Knol, Ruben Fernandez‐Santamaria, Joana Vitte, Elena Izquierdo, Cristina Gomez‐Casado, Marina Perez‐Gordo, Araceli Diaz Perales, Maria M. Escribese

**Affiliations:** ^1^ Department of Biology Faculty of Science Molecular Biology Section Hacettepe University Ankara Turkey; ^2^ Department of Biochemistry and Molecular Biology School of Chemistry Complutense University of Madrid Madrid Spain; ^3^ Allergy Unit and Research Laboratory Hospital Regional Universitario de Málaga IBIMA Plataforma BIONAND Málaga Spain; ^4^ Department of Clinical Medicine Aarhus University Aarhus Denmark; ^5^ Department of Respiratory Diseases and Allergy Aarhus University Aarhus Denmark; ^6^ Department of Dermatology and Allergy School of Medicine Technical University of Munich Munich Germany; ^7^ Center of Translational Immunology and Dermatology/Allergology University Medical Center Utrecht Utrecht the Netherlands; ^8^ Allergy and Immunology Department IIS‐Fundación Jiménez Díaz‐UAM Madrid Spain; ^9^ Immunology Laboratory University Hospital of Reims University of Reims Champagne‐Ardenne Reims France; ^10^ Department of Basic Medical Sciences School of Medicine Universidad San Pablo‐CEU Institute for Applied Molecular Medicine Nemesio Díez CEU Universities Boadilla del Monte Spain; ^11^ Institute of Applied Molecular Medicine Hospitals Madrid (HM) Group San Pablo‐CEU University Madrid Spain; ^12^ Department of Basic Medical Sciences Faculty of Medicine Institute of Applied Molecular Medicine‐Nemesio Díez (IMMA‐ND) Universidad San Pablo‐CEU CEU Universities Boadilla del Monte Spain; ^13^ Department of Biotechnology Center for Plant Biotechnology and Genomics (UPM‐INIA/CSIC) Universidad Politecnica de Madrid Madrid Spain

**Keywords:** allergy diagnosis, bioassays, endotype, precision allergy medicine, translational research

## Abstract

Bioassays offer functional readouts of immune responses through specific biomarkers, providing mechanistic insight beyond conventional serology. By capturing cellular activation, tissue‐level changes, and molecular signaling, they hold promise for refining allergy diagnosis, risk stratification, and treatment monitoring, contributing to precision medicine. This review summarizes the current landscape of three allergy‐relevant bioassays: basophil activation test, histology, and molecular biology‐based assays. We discuss their underlying principles, clinical utilities as well as methodological and translational challenges and translational barriers. Attention is given to unmet needs such as assay standardization, reproducibility, and integration into clinical workflows. Finally, we outline future directions for research and clinical implementation, emphasizing the potential of bioassays to refine personalized management of allergic disorders. This position paper clearly distinguishes between descriptive summaries of the available evidence and the potential applicability recommendations of the EAACI Task Force, thereby enhancing clarity and interpretability for clinicians and researchers.

## 1 Introduction

Allergy is a complex and heterogeneous condition that manifests in diverse ways and can affect several organs and systems [1]. Consequently, personalized management strategies are necessary to ensure accurate diagnosis and effective treatment. In this regard, it is essential to stratify patients into homogeneous groups according to their phenotypic and endotypic features [2]. This is only possible by identifying specific biomarkers and developing bioassays to detect them. Bioassays are analytical methods used to assess biological activity and functional immune responses in cells, tissues, and living systems [3]. In the field of allergy, bioassays provide functional assessment of immune responses through the evaluation of cellular activation, tissue‐level alterations, and biomarker‐associated molecular pathways, thereby supporting patient stratification and personalized diagnostic approaches. Based on their predictive accuracy and established clinical validation, certain bioassays have the potential to minimize the reliance on invasive or high‐risk diagnostic procedures, such as provocation tests, particularly in vulnerable patient populations [4].

At present, several highly valuable diagnostic strategies are available, such as the detection of serum‐specific immunoglobulin E (sIgE), assessment of cell phenotype, detailed clinical history, or provocation tests [5]. However, identifying reliable biomarkers for robust patient stratification and endotyping remains challenging. In many instances, particularly in allergy, such tools are either unavailable or not widely accessible. From a research perspective, numerous pioneering assays have been developed to quantify protein levels, assess cellular activation, evaluate tissue damage, and investigate other processes that are fundamental to understanding the mechanisms underlying allergic responses. However, these methodologies remain largely restricted to research settings and have not been integrated into routine clinical practice, with only limited applications in selected cases [6].

Despite advances in bioassay technologies, several challenges still limit their broader clinical implementation, including a lack of standardization, limited reproducibility across centers, regulatory barriers, accessibility, and insufficient integration into clinical workflows. Across all reviewed bioassay platforms, including BAT, histology, and molecular assays, harmonized protocols, validated biomarkers, and clinically applicable interpretation strategies remain essential for routine adoption in allergy practice [7, 8]. To facilitate broader clinical integration, standardized operating procedures and clinically applicable interpretation strategies remain important considerations.

Therefore, this position paper provides a narrative overview of selected bioassay methodologies used in allergy research, highlights current unmet needs, and outlines consensus‐based recommendations from the EAACI Task Force regarding their translational applicability in clinical practice. The recommendations presented in this position paper were developed through expert‐driven iterative discussions within the EAACI Task Force based on currently available evidence and collective clinical and translational research experience. The selected bioassay platforms were included based on their current relevance in allergy research, translational applicability, level of clinical integration, and representation in contemporary literature. Particular emphasis is placed on challenges related to standardization, automation, accessibility, reimbursement, guideline integration, and the need for clinically validated platforms.

## 2 Bioassays

### 2.1 Basophil Activation Test (BAT)

BAT is among the most clinically advanced functional bioassays in allergy diagnostics, particularly in drug allergy, Hymenoptera venom allergy, and selected food allergies. However, despite growing clinical adoption in specialized centers, broader routine implementation remains limited by issues related to standardization, automation, and accessibility. BAT may support patient‐tailored diagnosis by functionally assessing allergen‐specific cellular responses and identifying clinically relevant sensitization patterns in selected patients.

BAT is a functional test based on basophil activation. Upon allergen‐ or drug‐induced crosslinking of IgE antibodies bound to the high‐affinity IgE receptor (FcεRI) on the basophil surface, activation signals can be measured by flow cytometry (Figure 1). Activation may also occur from direct crosslinking of FcεRI by auto‐antibodies or ligand binding to G‐protein‐coupled receptors. This test uses flow cytometry to identify and measure basophil activation by tracking the increase in expression of CD63, CD203c, or both as activation markers. BAT results can be expressed as basophil reactivity as the percentage of CD63^+^ or CD203c^high^ cells among total basophils or basophil sensitivity when indicating the allergen concentration that triggers half‐maximal basophil activation, expressed as EC50 or CD‐sense. In cases of drug allergy, results can also be shown as a stimulation index (SI), representing the ratio of activated basophils after antigen stimulation to basophils activated spontaneously [9, 10].

**FIGURE 1:**
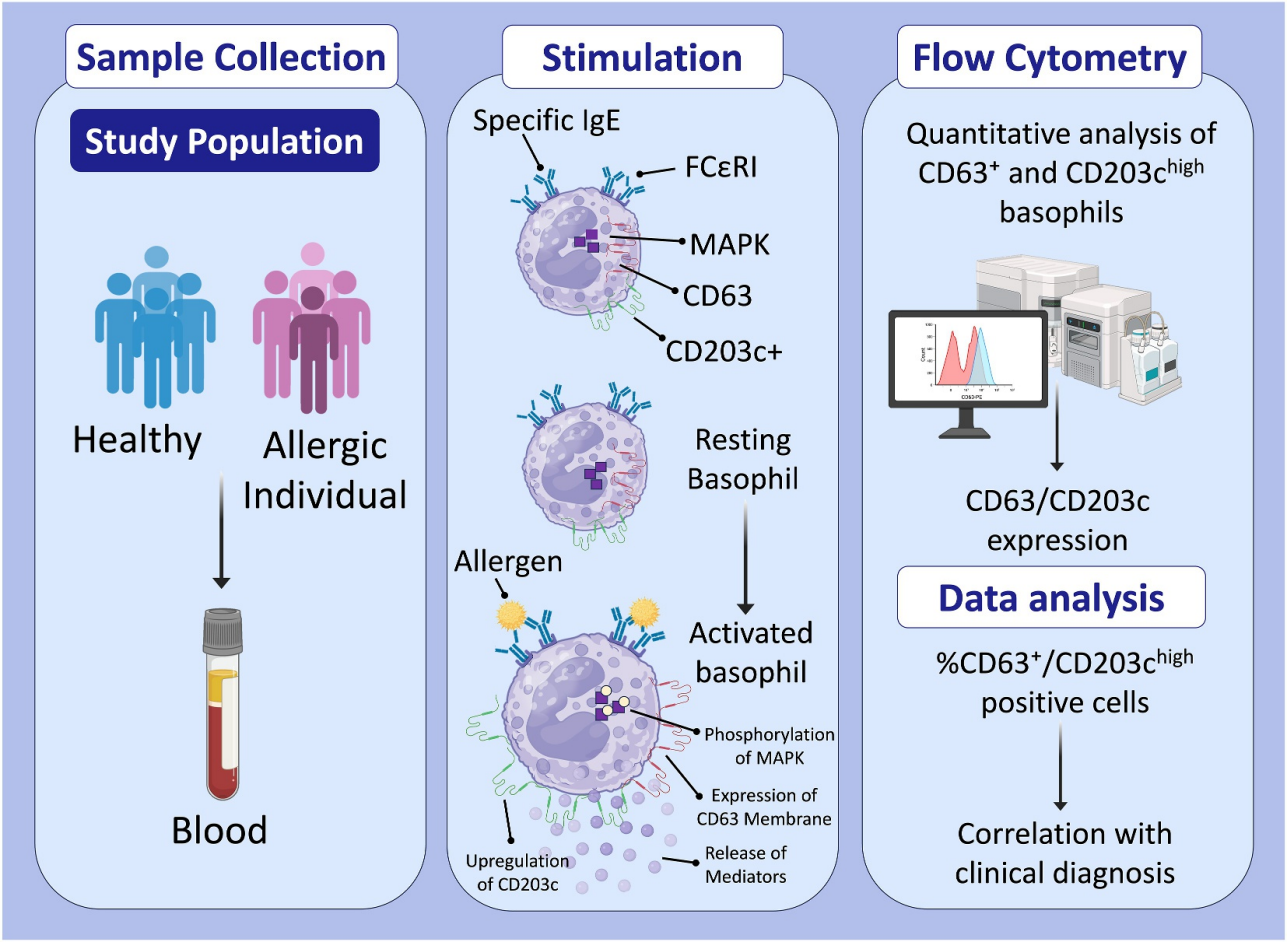
The basophil activation test (BAT) workflow. BAT is illustrated in a schematic diagram, showing sample collection, allergen‐induced basophil activation, and flow cytometric analysis of CD63/CD203c expression for correlation with clinical diagnosis. BAT, basophil activation test; FcεRI, high‐affinity IgE receptor; MAPK, mitogen‐activated protein kinase.

There are several applications of BAT to assess allergic reactions.

1. Diagnostic tool: BAT is useful for evaluating IgE‐mediated reactions in allergic rhinitis, asthma, food allergies, insect venom allergies, and drug allergies [9, 11, 12, 13]. Positive BAT results correlate more closely with clinical relevance than serum sIgE tests. It is particularly recommended when skin or serum sIgE tests are unavailable or unclear, and as a first step in life‐threatening drug allergies to avoid provocation tests. For chronic urticaria, BAT can detect autoreactive serum components.
2. Monitoring treatments: BAT is an appropriate bioassay for tracking the effectiveness of allergen‐specific immunotherapy (AIT), standard treatments, biological therapies, and rapid drug desensitization protocols [10, 14, 15].
3. Allergenicity assessment: BAT can assess the allergenic potential of various compounds or their modified forms. It can also detect small amounts of allergenic proteins at high sensitivity (∼1 ng/mL) in foods, which is critical in food allergies to avoid accidental exposure to allergens, even in low quantities [16, 17].

#### 2.1.1 Unmet Needs to Translate BAT Bioassays Into Clinics

BAT is often a more cost‐effective approach than provocation tests, taking into account the hospital facilities and personnel expertise and safety requirements associated with provocation procedures. It also has high diagnostic potential and is a noninvasive, reliable assay [9, 12]. However, several procedural limitations restrict its broader implementation. The method requires flow cytometry expertise as well as proper technical operation and maintenance. These requirements hinder its suitability as a point‐of‐care test, thereby limiting widespread adoption [18]. Additionally, reimbursement for BAT remains under discussion in many countries.

BAT can yield consistent, reproducible results when the same methods and strict standardization are followed. Indeed, when laboratories use a consensus protocol, they can show acceptable variability, with a coefficient of variation (CV) lower than 10% for detecting CD63^+^ basophils, as shown in ten European labs using preactivated blood [12, 19].

Variations in results can arise from differences in sIgE affinity or allergen epitope density, affecting the optimal allergen or drug concentration for basophil activation. The following points reflect consensus recommendations from the EAACI Task Force regarding translational priorities and methodological gaps.

1. Clinical validation: BAT is recognized as a clinical tool for diagnosing peanut and sesame allergies. Further validation across additional allergens is recommended to expand its diagnostic utility [11]. It is also recommended for evaluating drug allergies to beta‐lactam antibiotics and neuromuscular blocking agents, and as an initial step in the allergological assessment of patients who have experienced life‐threatening anaphylaxis to drugs, such as cardiac arrest, when drug provocation testing is contraindicated [9].
2. Standardization of experimental conditions [9, 11, 12]: Tests typically use whole fresh blood treated with anticoagulants, with EDTA being common in some commercial tests and heparin used in others. Clear definitions are needed for specific reagents and antibodies, whether they are fresh or freeze‐dried, including basophil and activation markers. The type of allergen (whole extract, components, native drugs, or metabolites) must be specified, along with the use of standardized allergens or drugs and the patient's current medication at the time of sample collection. To unify result analysis, the 2.5% threshold set for the negative population should be accepted [12].
3. To reduce the risk of basophil energy‐related false negatives, basophil activation testing is recommended no earlier than 3–4 weeks after the immediate reaction [9]. However, mast cells replenish secretory granules within 72 hours, and basophil granulocytes have a lifespan of under 2 days. There are reports of positive BAT results that align with positive skin prick tests and valid diagnoses.
4. BAT positivity can change over time, with activation declining after no further contact with the allergen or increasing after contact with the allergen [8].
5. In 10%–15% of patients, basophils may enter a temporary non‐release status that leads to invalid BAT results. This condition may vary based on the positive control used. It was recommended to retest nonresponders in BAT after 6 months to assess whether any modification occurs [9].

### 2.2 Histology

Histological approaches are widely used in both research and routine clinical practice, particularly in skin‐associated allergic diseases and chronic inflammatory airway disorders. Beyond diagnostic support, histology also contributes to disease endotyping, assessment of tissue remodeling, and evaluation of treatment‐associated tissue responses. Histological assessment may contribute to patient stratification by linking tissue‐level inflammatory patterns and remodeling features with disease endotypes and treatment‐associated responses.

Histology is the branch of biology that studies the composition, structure, and characteristics of the organic tissues in living organisms. The main histological techniques for examining microscopic tissue structures involve staining preserved tissue sections, either paraffin‐embedded or frozen, and using antibodies (immunohistochemistry (IHC)) or dyes (histological stains) [20]. Histological analysis is important because it helps evaluate disease mechanisms and therapeutic responses in situ (Figure 2). It provides direct evidence of tissue‐level immune responses in allergic inflammation, highlighting key aspects such as epithelial barrier dysfunction, tissue remodeling, immune cell infiltration, and structural tissue alterations in both mucosal and skin‐associated allergic diseases.

**FIGURE 2:**
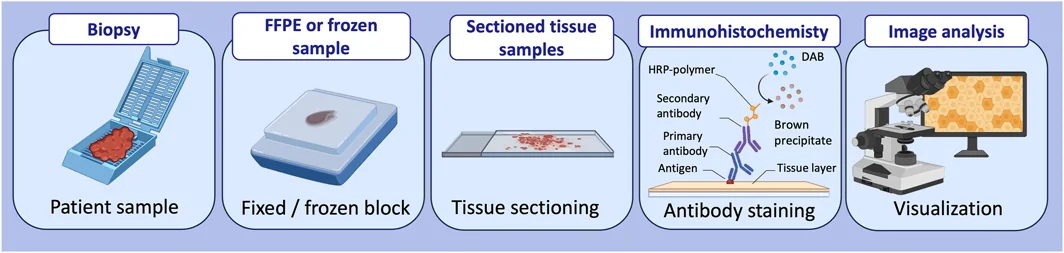
Schematic representation of histological staining and immunohistochemistry workflows. Schematic representation of histological staining and immunohistochemistry workflows. Biopsy samples are collected, preserved by formalin fixation and paraffin embedding (FFPE) or freezing, sectioned using a microtome, and mounted onto microscope slides for histological staining or immunohistochemistry. Image analysis, including digital and AI‐assisted approaches, may support semi‐quantitative evaluation of stained proteins of interest. DAB, diaminobenzidine; HRP, horseradish peroxidase.

In allergy research, histological methods assess epithelial barrier integrity, skin, vascular changes, connective tissue fibrosis, and the presence and distribution of immune cell infiltrates within tissues. Commonly analyzed samples include biopsies from the lung, skin, oral mucosa, and gut mucosa, which are crucial in allergic diseases. The detailed tissue‐level information provided by histological techniques help connect molecular mechanisms with clinical outcomes in allergy, offering insights into why some patients respond to specific treatments while others do not achieve tolerance [21, 22].

Additionally, histopathological analysis provides critical insights into tissue alterations and contributes to elucidating underlying disease mechanisms. They also enable direct assessment of tissue‐level changes after treatment, helping to monitor efficacy and disease progression over time [23, 24].

#### 2.2.1 Unmet Needs to Translate Histology Bioassays Into Clinics

While this technique is well established, several factors limit its reproducibility and routine clinical applicability, including variability in tissue section thickness, antigen retrieval methods, primary antibody specificity, and challenges with blocking nonspecific binding [25, 26]. Furthermore, histological techniques performed in conventional settings are often semi‐quantitative and may rely on observer‐dependent interpretation. However, recent advances in automated staining systems, digital pathology, and AI‐assisted image analysis have substantially improved reproducibility, standardization, and interpretation consistency across centers [27].

The invasive nature of biopsy collection, along with the need for specialized analysis, limits the technique's use for routine clinical monitoring or widespread screening. Histology can make a significant contribution to clinical research by validating biomarkers found through less invasive methods (e.g., blood or nasal swabs) and serving as a gold standard for tissue changes. Improvements in minimally invasive biopsy techniques combined with integration into digital and molecular pathology platforms could expand its use in clinical trials and future routine practice [27, 28, 29]. Increased standardization and integration of molecular pathology were identified by the EAACI Task Force as important priorities to enhance reproducibility and facilitate broader clinical application [29].

In summary, while histological techniques provide valuable information on disease processes, their integration into real‐time clinical decision‐making remains limited in allergy. In contrast, point‐of‐care histological assessment is well established in oncological disciplines, particularly in surgical oncology and dermato‐oncology, where intraoperative histological evaluation (e.g., frozen section analysis and Mohs micrographic surgery) is routinely used to support real‐time diagnostic and therapeutic decision‐making [30, 31]. Expanding such approaches to allergic diseases may represent a promising future direction, particularly in settings such as chronic rhinosinusitis with nasal polyps, where intraoperative tissue evaluation could inform disease endotyping and guide targeted interventions.

### 2.3 Molecular Biology and Immunology Assays

Molecular and immunology‐based bioassays encompass a broad spectrum of approaches ranging from nucleic acid detection to protein and biomarker profiling. While some assays are already integrated into specialized clinical diagnostic workflows, others remain primarily research‐oriented and require further validation before routine clinical implementation. These bioassays may support personalized allergy management through biomarker profiling, molecular endotyping, and identification of clinically relevant sensitization or immune response patterns. These approaches can broadly be divided into two main applications: (i) detection and characterization of allergens or allergen‐encoding sequences in environmental or food samples, and (ii) evaluation of patient‐derived molecular and immunological biomarkers associated with sensitization, inflammation, and disease endotypes [32].

Molecular biology assays, including DNA‐based techniques such as reverse transcription‐polymerase chain reaction (qPCR) and protein‐based techniques such as Western blot and enzyme‐linked immunosorbent assay (ELISA) have the potential to be used in the clinical diagnosis of allergic diseases (Figure 3). These assays can be used for diagnosis, detection of severity markers, therapy decisions, and clinical follow‐up. The objectives of allergy diagnostics include identification of clinically relevant sensitizations, immune dysregulation patterns, allergen triggers, and disease‐associated biomarkers.

**FIGURE 3:**
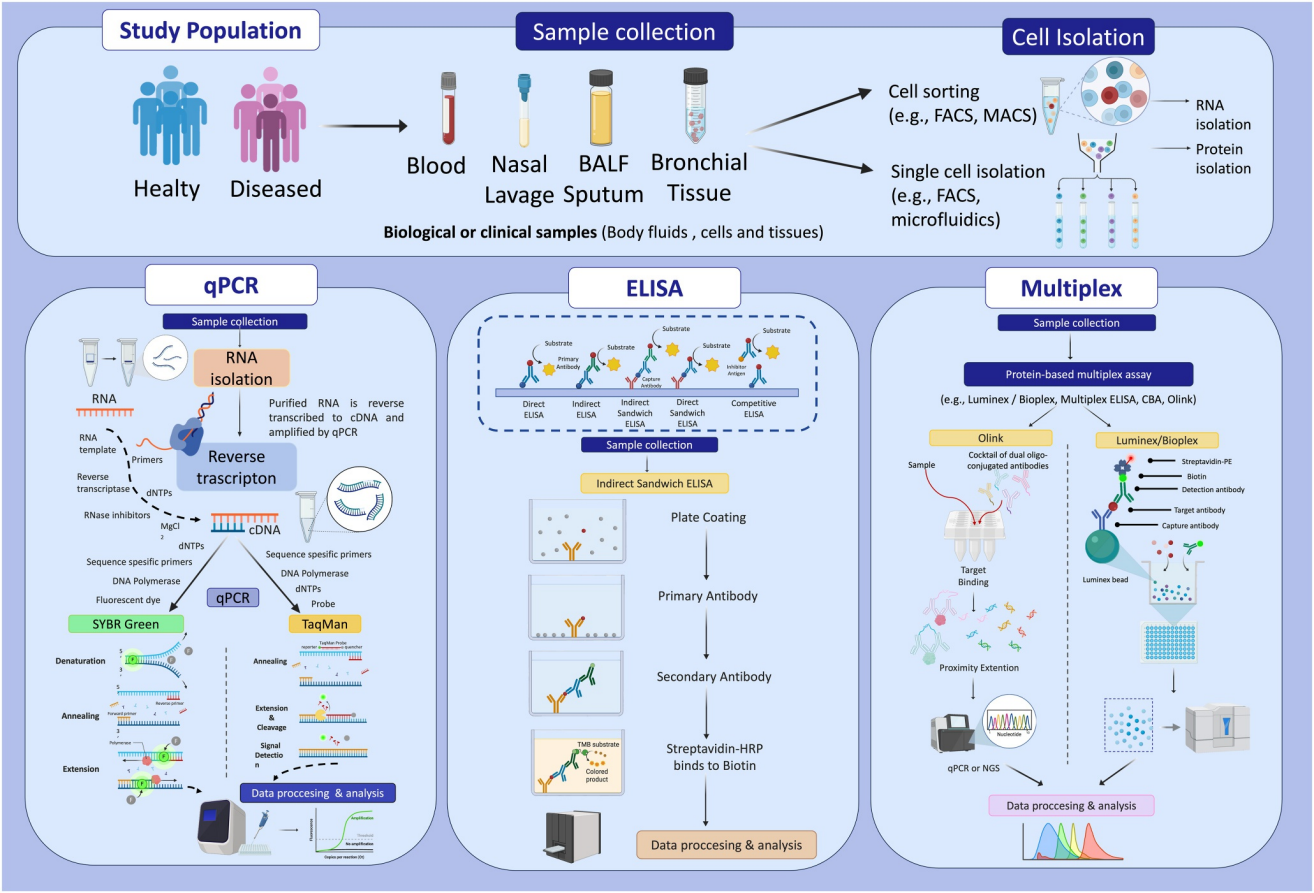
Graphical summary of major molecular biological assays used in allergy research and diagnostics. The figure illustrates study population and sample collection workflows, cell isolation approaches, qPCR‐based gene expression analysis, ELISA‐based protein detection, and multiplex assay platforms used for biomarker profiling and translational allergy research. BALF, bronchoalveolar lavage fluid; CBA, cytometric bead array; ELISA, enzyme‐linked immunosorbent assay; FACS, fluorescence‐activated cell sorting; HRPO, horseradish peroxidase; MACS, magnetic‐activated cell sorting; NGS, next‐generation sequencing; qPCR, quantitative polymerase chain reaction.

One of these molecular tools is qPCR, which is highly sensitive and specific, and allows quantitative evaluation of gene expression levels. It provides accurate and reproducible results even with minimal sample input and is ideal for detecting low‐abundance transcripts [32].

Western blotting is a commonly used protein analysis technique that enables evaluation of protein presence, molecular size, post‐translational modifications, and relative abundance across different biological conditions. It offers high specificity and enables visual confirmation of changes in protein synthesis under various experimental or clinical conditions. However, this technique is labor‐intensive, semi‐quantitative, and susceptible to variability due to antibody quality and transfer efficiency. While conventional Western blotting typically targets only one or a few analytes at a time and remains limited for broad‐scale biomarker profiling, modified immunoblot and line blot–based multiplex platforms are also used in selected clinical allergy diagnostic applications [33].

ELISA is also an effective quantitative protein detection tool in allergy research and diagnosis. It is relatively simple, cost‐effective, and offers good sensitivity and specificity for single‐analyte measurements, such as total or allergen‐specific IgE levels. However, traditional ELISA requires separate wells for each target, resulting in increased sample and reagent consumption. Although conventional ELISA formats are generally limited to single‐analyte detection, certain modified platforms allow partial multiplexing of a limited number of analytes. However, these approaches remain less suitable for large‐scale biomarker profiling compared with high‐throughput multiplex technologies [34].

Multiplex immunoassays, including the ImmunoCAP ISAC and ALEX2 platforms, enable simultaneous detection of sIgE antibodies against multiple allergen components with a minimal sample volume [35]. These approaches are particularly useful for polysensitized patients or in cases where serum availability is limited [36]. Today, omics‐based technologies, such as transcriptomics, proteomics, and metabolomics, are used in allergic disease research to identify new biomarkers and endotypes. These approaches provide a system‐level understanding of immune dysregulation, paving the way for personalized diagnostic and treatment strategies [37]. Although in vitro IgE assays remain valuable tools for supporting allergy diagnosis, their main limitation is their inability to distinguish sensitization from clinical relevance. Proper interpretation requires consideration of inter‐method heterogeneity and the continued need for careful interpretation of allergen‐specific reference ranges, particularly at low IgE levels.

#### 2.3.1 Polymerase Chain Reaction (PCR)

PCR is a widely used molecular technique that enables the exponential amplification of specific DNA sequences in a short time with high sensitivity. It involves repeated cycles of denaturation, annealing, and extension [38]. One of the most advanced examples of this method is qPCR, which quantifies DNA or RNA (including mRNA and miRNA) during amplification [39, 40]. qPCR is a preferred method for the rapid, sensitive, and specific detection and quantification of nucleic acids in various biological and environmental samples. qPCR is used in various applications, including gene expression analysis, genotyping, mutation detection, bioremediation monitoring, allelic discrimination, pathogen detection and quantification, and the identification of allergens in food products [39, 41, 42]. Moreover, qPCR can be used to identify allergen‐specific gene expression changes and profile immune‐related gene signatures in diseases such as asthma, food allergy, and allergic rhinitis [43, 44, 45]. In addition, multiplex qPCR allows for the detection of multiple targets in a single reaction, increasing efficiency in diagnostic assays [39, 41]. Overall, qPCR remains a fundamental tool in molecular biology owing to its high sensitivity, specificity, and versatility in various research and diagnostic contexts.

*Unmet Needs to Translate PCR‐Based Bioassays Into Clinics:* PCR is used to identify specific DNA fragments caused by point mutations, deletions, and insertions in disease‐associated genes. Although qPCR is not yet broadly implemented across all routine allergy diagnostic settings, selected qPCR‐based assays are already incorporated into specialized diagnostic algorithms in conditions such as Hymenoptera venom allergy, mastocytosis‐associated risk assessment, and hereditary alpha‐tryptasemia. In addition, qPCR plays an expanding role in food allergen detection and immune gene expression profiling [46, 47, 48].

qPCR has been applied to identify hidden allergenic components in foods, which is crucial for managing the risk of anaphylactic reactions in sensitized individuals [49] In clinical settings, qPCR is also used to monitor immune response not yet broadly implemented markers during allergen‐specific immunotherapy, such as tracking gene expression changes in children undergoing oral immunotherapy for egg or milk allergy, offering predictive value for treatment outcomes [50, 51]. Food allergen testing is essential to protect individuals with food allergies, which can range from mild symptoms to severe anaphylaxis. qPCR enables precise quantification of genes encoding allergenic proteins, supporting regulatory compliance and improving food safety standards [52]. However, detection of allergen‐encoding DNA sequences by PCR does not necessarily confirm the expression, presence, or biological activity of the corresponding allergenic proteins. Therefore, PCR‐based allergen detection should be interpreted together with protein‐based assays and clinical relevance [32, 53]. Thus, qPCR is evolving beyond its role as a molecular tool, contributing to allergy risk assessment, personalized management, and industrial quality control. However, its translation into routine allergy diagnostics remains limited because of the lack of well‐defined and validated molecular biomarkers specific to allergic diseases (29). Unlike transcriptome analyses, qPCR can measure only a limited number of gene expressions. Therefore, although broader routine implementation of qPCR‐based biomarker profiling in allergy practice remains variable, qPCR is increasingly contributing to translational research and specialized clinical diagnostics in allergy‐related diseases [54]. In addition, qPCR‐based approaches are widely used in clinical practice for the detection of infectious agents that may influence the severity of allergic diseases [55].

#### 2.3.2 Enzyme‐Linked Immunosorbent Assay (ELISA)

ELISA can be used to test for allergen‐specific IgE (sIgE) in patient sera. However, the presence of sIgE does not necessarily indicate a clinically allergic reaction. The severity of symptoms in an allergic individual is not fully correlated with or predicted by sIgE levels, but the likelihood of symptom onset is directly related [28].

Routine in vitro allergy diagnostics commonly use fluoroenzyme immunoassay (FEIA)‐based platforms for allergen‐specific IgE detection [56, 57]. These systems have improved singleplex testing strategies by enabling the use of allergen extracts and molecular allergen components for more detailed sensitization profiling [36].

Among the currently available platforms, ImmunoCAP is one of the most widely used systems in clinical allergy diagnostics [36, 58]. Nevertheless, immunoCAP is not available for all allergens and drugs, and with this method, only the detection of sIgE antibodies to specific allergens can be performed one at a time, thus requiring the selection of which allergens should be included in the test. Moreover, for drug allergy, sensitivity to available drugs, for example beta‐lactam antibiotics, is low [59]. In complex cases, such as those involving polysensitized patients with multiple positive skin prick test (SPT) results, the list of allergens and components that need to be tested can become quite extensive [32]. Nevertheless, the inclusion of selected molecular allergen markers, such as CCDs, PR‐10 proteins (e.g., Bet v 1 homologues), or profilins, may already provide clinically meaningful clarification in complex polysensitized patients by helping to distinguish cross‐reactivity patterns from genuine primary sensitizations [60, 61, 62]. In contrast, the most common use of ELISA in allergy is to measure the concentration of specific allergens in foods and other complex samples, and to correlate the quantity with the clinical outcome [63]. For this purpose, ELISA sensitivity must match clinically relevant thresholds (low‐dose exposure in highly sensitive individuals) [64]. Although there is no consensus on threshold doses for allergenic foods, the available clinical data indicate that the most sensitive individuals for peanut, milk, and egg exhibit mild symptoms at levels of 0.5–1 mg of the whole allergenic food [65, 66]. The threshold dose needed to trigger symptoms in individuals with IgE‐mediated food allergies is generally low; hence, sensitive ELISA analysis must be performed to detect even smaller quantities of potential allergens in the samples [67, 68].

*Unmet Needs to Translate ELISA‐Based Bioassays Into Clinics:* ELISA requires optimization before performing the assay. Key parameters to consider include antibody concentration, incubation time, buffers, substrate solution, and wash solution. One of the main objectives is to carefully select and validate capture and detection antibodies, as well as to determine the most suitable detection mode to achieve the highest sensitivity and specificity. It is also necessary to overcome nonspecific binding, interferences, and matrix effects, as previously noted. Background noise must be considered. For example, indirect detection can yield higher signals and may therefore be more sensitive, but it may also increase background signal, thereby reducing specificity. Achieving an appropriate balance is essential for developing the most suitable ELISA for each experimental context. This flexibility allows for the optimization of parameters to produce a highly sensitive assay. Practical integration into clinical workflows will require harmonized assay validation criteria, standardized reference materials, and clearer interpretation guidelines for clinicians.

#### 2.3.3 Multiplex Assays

Multiplex assays are powerful tools that enable the simultaneous quantification of multiple analytes in a single, small‐volume biological sample. These assays require minimal sample preparation and can be applied to a wide range of sample types, including plasma, serum, bronchoalveolar lavage fluid (BALF), induced sputum, tissue lysates, saliva, urine, and cell culture supernatants [69, 70]. Multiplex assays are used for various purposes including biomarker validation, allergy screening, protein profiling, pathway analysis, and clinical diagnostics.

In allergy diagnostics, multiplex assays enable the detection of sIgE to multiple allergens or allergen components in a single test. Common platforms include multiplex assays such as ImmunoCAP ISAC or ALEX system [71, 72]. These tools provide detailed IgE profiles, which are particularly useful in complex cases such as polysensitized patients [73].

Although multiplex assays are increasingly explored and used in allergy research and selected clinical settings, several limitations still affect their broader implementation in routine practice [5, 74]. With the development of molecular diagnostics, recombinant or purified allergens have improved the sensitivity and specificity of singleplex assays. Still, not all molecular allergens are currently available for routine testing.

Beyond allergy, multiplex protein profiling is essential in monitoring cytokine and chemokine levels in multifactorial diseases [75]. In addition to IgE profiling, recent advances in multiplex proteomics, particularly the Olink proximity extension assay (PEA) technology, use minimal sample volumes (1 μL/plasma, serum, etc.) [76, 77]. Olink is well‐suited for profiling cytokines, chemokines, and growth factors implicated in allergic inflammation, epithelial dysfunction, and immune dysregulation [78]. Similarly, Luminex xMAP technology employs color‐coded microsphere beads coupled with specific antibodies, enabling simultaneous quantification of up to 500 analytes in a single sample [79, 80]. Several multiplex and high‐throughput proteomic platforms are increasingly being explored for immune profiling, biomarker discovery, and translational allergy research, particularly in pediatric and precision medicine settings. These technologies are increasingly being investigated in combination with data‐driven analytical approaches to support biomarker discovery, patient stratification, and translational research in allergy and other immune‐mediated diseases [37, 81, 82].

*Unmet Needs to Translate Multiplex Bioassays Into Clinics:* Multiplex bioassays present challenges to clinical interpretation despite their potential. Platforms such as Luminex (Bio‐Plex) and Olink enable simultaneous quantification of multiple biomarkers, offering powerful tools for immune profiling, endotyping, and biomarker discovery in allergic diseases [83, 84, 85]. However, the interpretation of these complex datasets remains a key barrier to clinical translation. The distinction between sensitization and clinically relevant allergy applies to both singleplex and multiplex allergen‐specific IgE assays. However, multiplex platforms may increase the likelihood of detecting incidental or clinically irrelevant sensitizations because of their broad allergen coverage, thereby increasing the complexity of clinical interpretation in the absence of compatible clinical history or confirmatory testing [86, 87]. Positive sensitization findings without compatible clinical history or confirmatory testing should be interpreted cautiously, although in selected clinical contexts such as allergen immunotherapy follow‐up or high‐risk patients in whom provocation testing is contraindicated, these findings may still provide clinically relevant information [87, 88]. Platform‐specific limitations further complicate their use in clinical settings. Luminex‐based assays can be affected by bead cross‐reactivity and signal overlap, requiring careful panel design and standardization [89]. Olink provides high sensitivity and specificity but often lacks validated clinical cutoffs, making interpretation context‐dependent. Cross‐reactivity, carbohydrate cross‐reactive determinants (CCDs), and differences in allergen or protein abundance introduce additional complexity [90, 91]. Multiplex results may also indicate unexpected sensitizations that are not clinically relevant, especially in the absence of corroborating patient history [92]. This is particularly applicable to insect venom hypersensitivities and food‐pollen syndromes, where immune responses may not align with clinical symptoms [5, 93]. Moreover, geographical variations in allergen profiles and severity indicators, such as lipid transfer proteins (LTPs) and Bet v1 homologues, highlight the need to support region‐specific optimization of multiplex allergen panels [5, 94]. Clinical decisions must therefore be based on a comprehensive patient history and confirmed allergic reactions, not sensitization or biomarker shifts alone [4].

## 3 Future Perspective

Emerging technologies such as microfluidics, nanotechnology, and automated high‐throughput platforms may improve the sensitivity, specificity, and scalability of bioassays in allergy research and clinical practice [95, 96, 97]. This will enable quicker, more accurate detection of allergic responses. These advancements may facilitate the development of point‐of‐care and workflow‐compatible bioassays that could support faster diagnostic and monitoring strategies in selected clinical settings which can be performed at the patient's bedside or in a clinician's office, reducing the time needed for diagnosis and treatment initiation.

Another important focus is integrating bioassays with other diagnostic tools and clinical data to create detailed patient profiles. By combining bioassay results with information about a patient's history, environmental exposures, and genetic risks, clinicians may be able to design personalized treatment plans that meet the unique needs of each patient. This approach aligns with precision medicine and may contribute to improved patient stratification and disease management.

However, several challenges must be tackled to support broader clinical implementation of bioassays in allergy clinical management. One challenge is the need to standardize and validate bioassay methods. As new bioassays emerge, it is crucial to establish standardized protocols and quality control measures within multicentric studies to ensure reliability and accuracy across different laboratories and clinical settings. Regulatory approvals and guidelines will also be vital to encourage widespread use of bioassays in clinical practice. To support clarity, future recommendations should continue distinguishing between narrative review content and consensus‐based guidance to facilitate clinical translation.

Moreover, education and training for healthcare professionals will be key to ensuring they use and interpret bioassay results correctly. Clinicians must understand the different types of bioassays, their limitations, and how to incorporate them into their clinical decisions (Table 1). Public awareness campaigns can also help raise understanding and acceptance of bioassays among patients and the public. A critical next step is the development of workflow‐ready tools such as automated data interpretation pipelines, interoperable reporting formats, and integration with electronic health records to ensure that bioassay results can be more effectively incorporated into routine clinical decision‐making.

**TABLE 1:** Summary of advantages and limitations of key bioassay platforms in allergy.

Bioassay	Main advantages	Main Limitations/unmet needs
Basophil activation test (BAT)	Functional readout of IgE‐mediated activation	Limited availability and reimbursement variability
	High specificity; correlates well with clinical relevance	Technically demanding and labor‐intensive
	Useful when SPT/sIgE are inconclusive or contraindicated	Requires flow cytometry expertise
	Valuable for monitoring AIT, biologics, and desensitization	Lack of full standardization across labs
	Enables allergenicity assessment of foods, drugs, and novel compounds	Requires fresh blood
	Reduces the need for provocation tests	Temporary non‐responder basophils in 10%–15% of patients
	Safe (no risk for the patient)	Not yet suitable as a point‐of‐care test
Histology/Immunohistochemistry	Direct visualization of tissue‐level inflammation	Semi‐quantitative; observer‐dependent unless digitized
	Essential for mechanistic studies and biomarker validation	Limited use for routine monitoring
	Increasing integration with digital pathology and AI	Variability in staining, antigen retrieval, and antibody specificity
	Supports disease endotyping	Requires specialized pathology infrastructure
PCR‐based assays (qPCR, multiplex PCR)	Highly sensitive and specific nucleic acid detection	Requires high‐quality nucleic acids
	Useful for gene expression profiling and immune signatures	Limited number of targets per reaction
	Applicable to food allergen detection and risk assessment	Biomarker panels for allergy not yet validated for routine care
	Increasing relevance in mastocytosis, HaT, and immunotherapy monitoring	Reflects transcription, not protein function
	Supports biomarker identification	Not widely implemented in standard allergy clinics
ELISA (singleplex protein assays)	Quantitative, robust, and widely available	One analyte per well → high sample/reagent use
	High sensitivity for allergen quantification in foods	Limited multiplexing capability
	Useful for cytokines, IgE, IgG4, and biomarker studies	Background noise and matrix effects require optimization
	Flexible assay optimization	Interpretation depends on validated reference ranges
Multiplex assays (ISAC, ALEX2, luminex, olink)	Comprehensive IgE or cytokine profiling from minimal sample volume	Complex data interpretation; sensitization ≠ clinical allergy
	Ideal for polysensitized patients and endotyping	Platform‐specific variability and lack of harmonized cut‐offs
	Enables systems‐level biomarker discovery	Higher cost and limited availability
	Efficient for pediatric and low‐volume samples	Risk of incidental findings requiring expert interpretation
	Supports precision medicine approaches	Not all allergens or biomarkers are represented

Abbreviations: AIT, allergen immunotherapy; HaT, hereditary alpha‐tryptasemia; SPT/sIgE, skin prick test/specific immunoglobulin E.

Overall, bioassays could increasingly contribute to precision allergy medicine as advances in validation, standardization and clinical translation are still continuous. Ongoing research, multicenter collaboration, and technology development will continue to be needed to support the broader use of bioassay‐based approaches in allergy and patient management.

## Author Contributions

Maria M. Escribese and Oscar Palomares designed the manuscript structure. Cagatay Karaaslan, Oscar Palomares, Busra Kilic, Cristobalina Mayorga, Hans Jürgen Hoffmann, Bernadette Eberlein, Edward Knol, Ruben Fernandez‐Santamaria, Joana Vitte, Elena Izquierdo, Cristina Gomez‐Casado, Marina Perez‐Gordo, Araceli Diaz Perales, Maria M. Escribese contributed to writing the manuscript sections. Cagatay Karaaslan, Araceli Diaz Perales and Maria M. Escribese organized the manuscript, completed the text, Busra Kilic and Cagatay Karaaslan performed the figures.

## Ethics Statement

This position paper is based on previously published, publicly available scholarly articles and does not include any original data.

## Conflicts of Interest

Cagatay Karaaslan, Busra Kilic, Maria M. Escribese, Ruben Fernandez‐Santamaria, Joana Vitte, Cristobalina Mayorga, Bernadette Eberlein, Edward Knol, Elena Izquierdo, Cristina Gomez‐Casado, Marina Perez‐Gordo, Araceli Diaz Perales declared no COI. Hans Jürgen Hoffmann is founder of AITSU ApS, with no relevance to this work. Oscar Palomares received research grants from MINECO, Ministerio de Ciencia, Innovación y Universidades, CAM, Inmunotek S. L., Novartis, and AstraZeneca and fees for giving scientific lectures or participation in Advisory Boards from: AstraZeneca, Pfizer, GlaxoSmithKline, Inmunotek S. L, Novartis, Sanofi‐Genzyme and Regeneron. All authors have read and approved the manuscript.

## Data Availability

The data that support the findings of this study are available on request from the corresponding author.
